# Stability Study of Meropenem 50 mg/mL Eye Drops in Polypropylene Dropper Bottles

**DOI:** 10.3390/pharmaceutics18080971

**Published:** 2026-08-07

**Authors:** Juan Carlos Ruiz Ramirez, María Encarnación Martínez Madrid, Adrián Gómiz Sáez, Alice Charlotte Viney, José María Alonso Herreros, Pilar Almela Rojo

**Affiliations:** 1 Pharmacy Service, Los Arcos del Mar Menor University General Hospital, Murcian Health Service, San Javier, 30739 Murcia, Spain; alice.charlotte@carm.es (A.C.V.); josem.alonso@carm.es (J.M.A.H.); 2 Department of Pharmacology, Faculty of Pharmacy, CEIR Campus Mare Nostrum (CMN), University of Murcia, 30120 Murcia, Spain; palmela@um.es; 3 Biomedical Research Institute of Murcia Pascual Parrilla—IMIB, 30120 Murcia, Spain; 4 Pharmacy Service, Santa Lucía University General Hospital, Murcian Health Service, Cartagena, 30202 Murcia, Spain; mariae.martinez48@carm.es; 5 Pharmacy Service, Virgen de la Salud—Elda General Hospital, Valencian Health Service, Elda, 03600 Alicante, Spain; gomiz_adrian@gva.es

**Keywords:** meropenem, ophthalmic solution, stability, freezing, polypropylene, HPLC, hospital compounding

## Abstract

**Background/Objectives:** Meropenem is a broad-spectrum carbapenem antibiotic with demonstrated efficacy against multidrug-resistant Gram-negative pathogens. Although its use as an ophthalmic formulation is off-label, growing clinical evidence supports its application in severe ocular infections such as keratitis and endophthalmitis. However, the intrinsic instability of meropenem in aqueous solutions and the absence of standardized ophthalmic preparations limit its routine use. Furthermore, no stability studies are currently available for meropenem 50 mg/mL eye drops stored in polypropylene (PP) dropper bottles under freezing and subsequent refrigerated conditions. The aim of this study was to evaluate the physicochemical and microbiological stability of a 50 mg/mL meropenem ophthalmic solution prepared in a hospital pharmacy using a commercial meropenem pharmaceutical product, and packaged in PP containers. **Methods:** Eye drops were aseptically prepared from a commercially available pharmaceutical product, containing 1g de meropenem and anhydrous sodium carbonate as an excipient. After preparation, the drops were stored at −20 ± 2 °C for up to 42 days, followed by refrigerated storage (5 ± 3 °C) after thawing for up to 7 days. Chemical stability was assessed using a validated stability-indicating HPLC method in accordance with ICH guidelines and was defined as 90–110% recovery of the initial concentration. Physical stability (appearance, pH, particulate matter) and microbiological stability were also evaluated under simulated in-use conditions. **Results:** The HPLC method demonstrated excellent linearity, precision, and accuracy. Meropenem concentrations remained within the predefined acceptance limits throughout the 42-day study period under freezing conditions, with no significant changes in pH, color, or particulate formation. After thawing, a progressive decrease in drug concentration was observed under refrigerated conditions, falling below 90% of the initial concentration within 24–48 h. A concomitant color change from colorless to yellow was also detected, consistent with β-lactam ring hydrolysis. Despite this degradation, no significant changes in physical parameters other than color were observed, and microbiological testing confirmed sterility for up to 7 days under refrigerated conditions. **Conclusions:** Meropenem drops 50 mg/mL in PP dropper bottles are physicochemically and microbiologically stable for 43 days (42 days under frozen conditions plus 1 day, in-use conditions, after opening and under refrigeration).

## 1. Introduction

**Figure 1:**
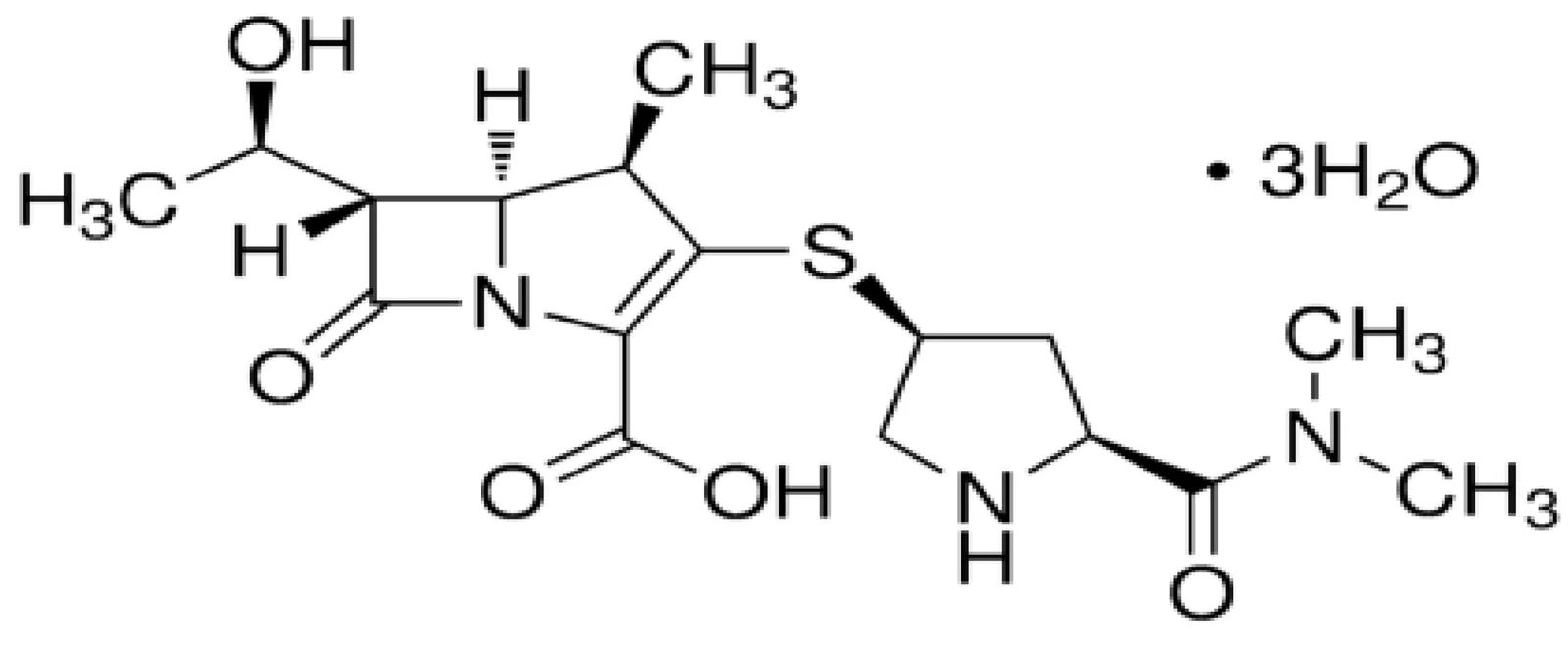
Structure of meropenem trihydrate.

Meropenem trihydrate—(4R,5S,6S)-3-[(3S,5S)-5-(dimethylcarbamoyl)pyrrolidin-3 -yl]sulfanyl-6-[(1R)-1-hydroxyethyl]-4-methyl-7-oxo-1-azabicyclo[3.2.0]hept-2-ene-2-carboxylic acid trihydrate (Figure 1)—is a white or almost white (or light yellow) crystalline powder, slightly soluble in water and practically insoluble in ethanol and dichloromethane [1].

Meropenem is a carbapenem antibiotic that exerts bactericidal activity through the inhibition of bacterial cell wall synthesis in both Gram-negative (G−) and Gram-positive (G+) organisms. Its antimicrobial spectrum encompasses a wide range of aerobic and anaerobic G− bacilli and G+ cocci. Notably, meropenem retains activity against aerobic G− bacilli that produce extended-spectrum β-lactamases (ESBLs) [2,3].

In Spain, meropenem is marketed as a powder to be used in solution for injection or infusion in vials containing 500 mg or 1000 mg of the active pharmaceutical ingredient (API) and anhydrous sodium carbonate (ASC) as the excipient. The ASC present in the meropenem formulation serves a technological function as an alkalinizing agent, intended to improve the stability of the active ingredient and maintain appropriate physicochemical conditions in the lyophilized product. It appears to be the standard excipient in commercial injectable meropenem formulations worldwide, and no evidence has been found of another commercial formulation using a different excipient. For intravenous administration, it must be reconstituted with water for injection (WFI), in the case of administration as an intravenous bolus injection, or with 0.9% aqueous sodium chloride solution (NS) or 5% aqueous dextrose solution (D5W) prior to dilution in NS or D5W for administration by intravenous infusion [2].

Meropenem is indicated for the treatment of the following infections in adults and children over 3 months of age: severe pneumonia, including hospital-acquired pneumonia and ventilator-associated pneumonia; bronchopulmonary infections in cystic fibrosis; complicated urinary tract infections; complicated intra-abdominal infections, intra- and postpartum infections; complicated skin and soft tissue infections; and acute bacterial meningitis [2]. Meropenem is not indicated for the treatment of ocular infections such as keratitis and endophthalmitis in its Summary of Product Characteristics; therefore, its use in these indications would be considered an off-label use, according to Spanish legislation [4]. However, the use of meropenem in ocular infections is supported by preclinical studies, animal models, clinical cases, and small case series, in which it has demonstrated efficacy and safety [5,6] for the treatment of severe keratitis caused by multidrug-resistant G− microorganisms [6,7,8,9,10,11], and in endophthalmitis due to resistant pathogens [12,13,14], as well as a demonstrated feasibility and favorable pharmacokinetics [7], including adequate penetration into ocular tissues [7,14,15] and a low toxicity profile [5,7], when administered both systemically and as ophthalmic eye drops. Nevertheless, the short stability of meropenem eye drops—24 h for an opened bottle and 48 h for a closed bottle—together with the cost of frequent preparation, limits their routine use in the aforementioned eye infections [9].

When formulating a meropenem eye drop solution at 50 mg/mL, it is essential to consider several critical formulation parameters. The physicochemical and microbiological stability of an ophthalmic solution depends on a series of interrelated factors that affect both the concentration of the active ingredient and the formulation medium, as well as the storage environment. For this reason, stability is usually expressed as a specific shelf life (for example, X days at 2–8 °C, either in closed or opened containers) and is experimentally defined by monitoring parameters such as concentration, pH, osmolality, organoleptic characteristics, and absence of microbial growth [16,17,18]. However, it should be noted that the aforementioned parameters are not an exclusive list for establishing stability, but rather represent examples of possible parameters that may be used among others [19].

From a stability perspective, meropenem is highly susceptible to degradation both in aqueous solutions and in its solid state due to the significant intrinsic strain of its fused ring system [20]. Its degradation pathways involve multiple chemical interactions in which different structural moieties of the drug participate. The main degradation mechanisms are: β-lactam ring hydrolysis, which is the principal degradation pathway of the drug in aqueous solutions and the primary mechanism by which enzymes such as β-lactamases inactivate the antibiotic [21,22] (the hydrolysis of this ring can be observed as a shift to a yellowish coloration [23,24]); intermolecular aminolysis, which is the fundamental cause of concentration-dependent degradation that occurs when the meropenem molecule degrades itself through a nucleophilic attack in which the side chain of one molecule attacks and cleaves the carbonyl of the β-lactam ring of a neighboring second meropenem molecule [21,25], thus also producing the aforementioned color change; inactivation of meropenem by intramolecular cyclization of the molecule with formation of a β-lactone, which is mediated by certain enzymatic targets (such as class D serine β-lactamases or l,d-transpeptidases) [22]; and thermal degradation and decarboxylation of meropenem in the solid state when subjected to high temperatures, which is due to decarboxylation and aromatization of the pyrrolidine ring (concomitant with hydrolysis of the β-lactam ring), yielding a by-product named 4-methyl-3-(1H-pyrrol-3-ylsulfanil)-5H-pyrrol-2-carboxylic acid [20,26].

Another issue to consider is the type of container that will hold the preparation. In this study, a PP dropper bottle was chosen. PP is widely regarded as one of the most inert plastics, exhibiting a low tendency to adsorb drug substances. In practice, adsorption to PP is generally negligible; however, it may become significant for drugs at low concentrations or with protein/peptide-like characteristics, as well as for high-value agents such as certain radiopharmaceuticals or insulin. Several studies have demonstrated that the physicochemical stability of most drug formulations is preserved in PP packaging, with no appreciable degradation observed over storage periods ranging from several days to weeks, depending on the specific compound and storage conditions (e.g., refrigeration, protection from light). PP displays low permeability to water vapor and is relatively impermeable to oxygen, which contributes to reduced degradation by oxidation or hydrolysis. Nevertheless, its gas barrier properties are less hermetic than those of glass; therefore, in long-term storage scenarios, the shelf life of particularly sensitive solutions may be compromised. Pharmaceutical-grade PP packaging is specifically designed to minimize particulate contamination and the migration of additives, thereby supporting both product quality and patient safety [27,28,29].

Currently, there are no stability studies on a meropenem 50 mg/mL ophthalmic solution, packaged in PP dropper bottles, under freezing and refrigerated conditions. For this reason, the present study investigates the physicochemical and microbiological stability of such a preparation.

## 2. Materials and Methods

### 2.1. Preparation of Meropenem 50 mg/mL Ophthalmic Solution

**Figure 2:**
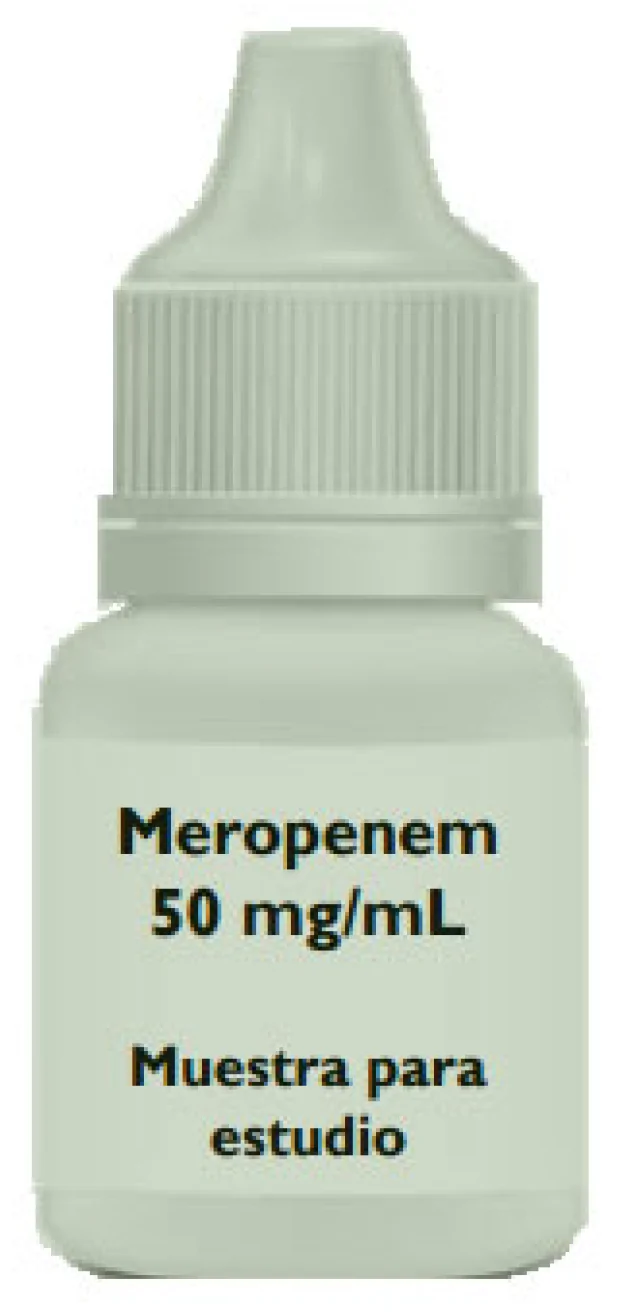
Dropper bottle containing 4 mL of ophthalmic solution.

The preparation process was carried out by reconstituting each 1 g meropenem vial (Aurovitas Spain, S.A.U., Madrid, Spain), which contains anhydrous sodium carbonate as an excipient, with 20 mL of WFI (Serra Pamies, S.A., Reus, Spain), in accordance with the specific recommendations for sterile preparations included in the Guide for good preparation practices of medicines in hospital pharmacy services (GPPMHPS) [30]. The contents of each reconstituted vial were transferred to a sterile, pyrogen-free vial (VacuflascTM, Grifols Movaco, S.A., Barcelona, Spain) until a total volume of 420 mL of the ophthalmic solution was obtained. Then, 4 mL of that solution was dosed into 90 sterile white PP dropper bottles of 10 mL of volume with photoprotective treatment (Oiarso S. Coop., Hernani, Spain) (Figure 2).

### 2.2. Chemical Stability

The eye drops were divided into two groups: frozen stability (FSG) and refrigerated stability after thawing (RSG), in which the eye drop bottles belonging to the FSG were thawed on the day of analysis and moved to the RSG, to be analyzed on the days established according to the analysis protocol. The freezing conditions were −20 ± 2 °C and the refrigeration conditions were 5 ± 3 °C. The thawing was carried out rapidly, by removing the eye drops from the freezer and waiting until they reached room temperature (1 h).

**Figure 3:**
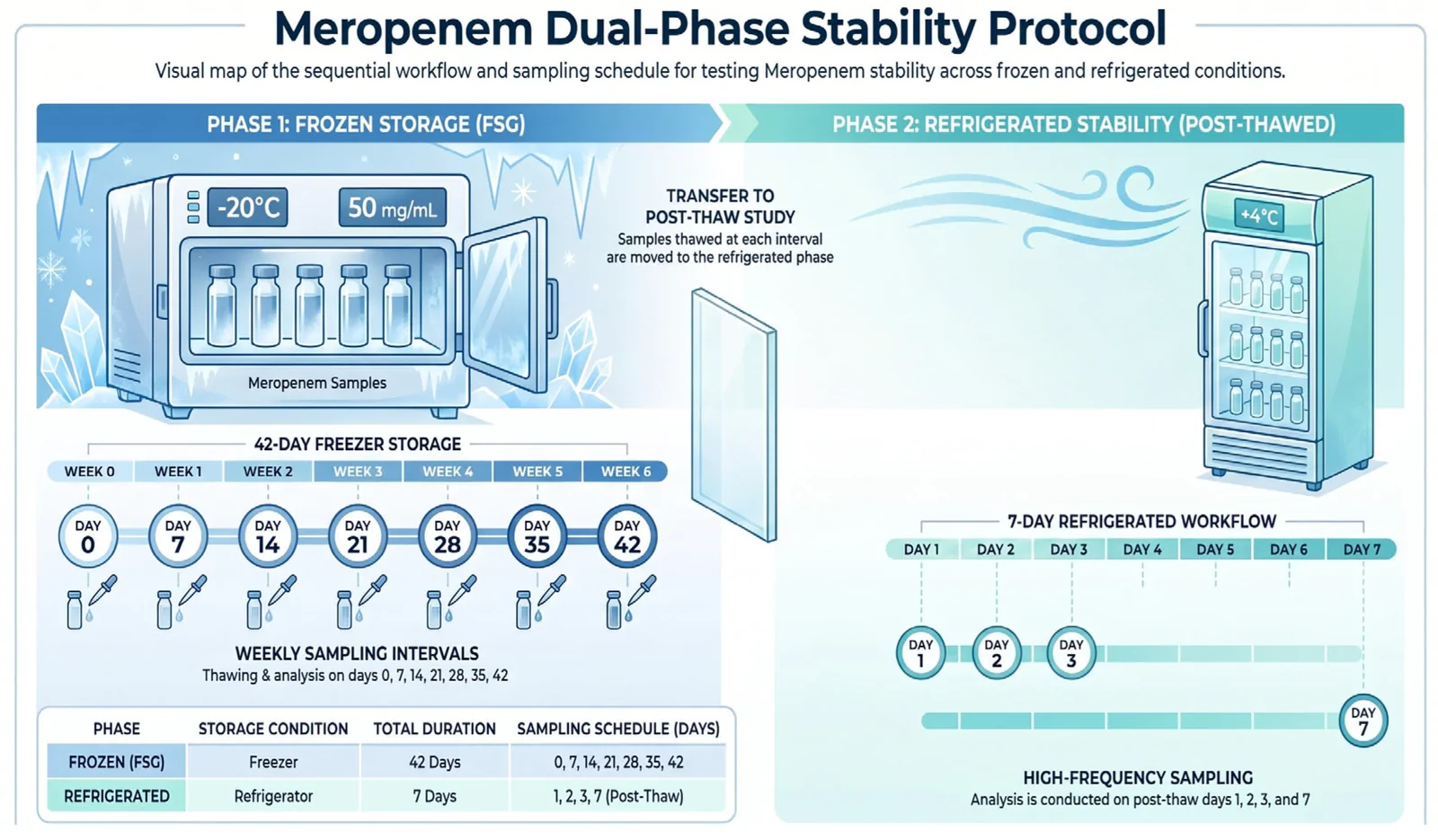
Study design scheme.

The chemical stability of meropenem 50 mg/mL ophthalmic solution in the FSG was studied over 42 days of storage: days 0 (D0), 7 (W1), 14 (W2), 21 (W3), 28 (W4), 35 (W5) and 42 (W6); and over 7 days of storage in the RSG after thawing, days 1 (WnD1), 2 (WnD2), 3 (WnD3) and 7 (WnD7), where “n” is the number of weeks during which the eye drops remained frozen and, after thawing, were transferred to the refrigerated study (Figure 3).

The preparation was considered stable if drug concentration remained within 90–110% of the initial concentration throughout the 42 days in the FSG and 7 days in the RSG [31,32,33].

### 2.3. Chromatographic Method

A Waters Breeze HPLC system (Waters Cromatografía, S.A., Barcelona, Spain) equipped with a XBridge 5 µm C18 reversed-phase column (130 Å pore size, 4.6 × 150 mm; Waters Cromatografía, S.A., Barcelona, Spain) was used for the study. Chromatographic separation was carried out under isocratic conditions using a mobile phase consisting of ultrapure water/acetonitrile/methanol (50/30/20, *v*/*v*), adjusting the pH to 7.5 with 10% *v*/*v* phosphoric acid. The flow rate was 1 mL/min, with detection at 300 nm, a column temperature of 25 °C, an injection volume of 20 µL, and a total run time of 2 min [34]. HPLC-grade acetonitrile, methanol and phosphoric acid were purchased from Panreac Química S.L.U. (Barcelona, Spain), and ultrapure water was obtained using the Milli-Q^®^ Integral 3/5/10/15 system (Merck KGaA, Darmstadt, Alemania). The meropenem reference standard was obtained from Merck Life Science, S.L. (Madrid, Spain).

Method validation: The HPLC method was validated for linearity, precision, and accuracy in accordance with the International Council for Harmonization of Technical Requirements for Pharmaceuticals for Human Use (ICH) Q2(R1) guidelines [35]. Linearity between the peak area and the meropenem concentration was assessed across six calibration levels from 0 to 150 mg/mL (0, 15, 30, 60, 90, 120, and 150 mg/mL). A calibration curve was constructed with linear regression analysis to determine the coefficient of determination (R^2^), slope (a), and y-intercept (b) [33]. Precision was evaluated through intra- and inter-day repeatability studies, keeping the samples refrigerated (2–8 °C) to preserve their stability during the assay. The intra-day assessment involved six replicate analyses on the same day at 80%, 100%, and 120% of the target concentration (50 mg/mL), while the inter-day study comprising three replicates over five different days at the same relative concentrations. Mean, standard deviation, and coefficient of variation were calculated, with acceptance criteria of <1% for intra-day and <2% for inter-day repeatability [33,34]. Accuracy was determined via recovery studies performed in triplicate at meropenem concentrations of 40, 50, and 60 mg/mL. Recovery percentages were calculated and compared to the theoretical value (100%) using Student’s *t*-test [33,34,36]. The limit of detection (LOD) and limit of quantification (LOQ) for meropenem were calculated using the standard deviation of the response (σ) and the slope of the calibration curve (S) from the calibration curve, applying the equations LOD = 3.3 × σ/S and LOQ = 10 × σ/S as per ICH recommendations [33,35,37].

This chromatographic method was validated for the quantification of intact meropenem under the chromatographic conditions employed, based on a previously published stability-indicating study in which meropenem samples were subjected to forced degradation under thermal, photolytic, acidic, alkaline, and oxidative conditions [34]. However, dedicated forced-degradation experiments to formally demonstrate the absence of interference from degradation products were not performed in this study; therefore, on each day of analysis, a meropenem standard solution (50 mg/mL) was injected in triplicate. Quantification of meropenem in both the standard and the sample was performed using an external standard calibration curve. Peak identity was confirmed by retention time matching, and the sample concentration was determined by interpolation on the calibration curve, allowing estimation of the percentage of remaining meropenem in the sample.

### 2.4. Physical Stability

Physical stability assessments comprising macroscopic visual inspection, pH and osmolality measurements, and microscopic evaluation for crystallization. On designated evaluation days, 1 mL aliquots from each formulation were withdrawn and subjected to detailed visual examination for anomalies including particulate matter, crystalline precipitates, turbidity, sedimentation and color alterations throughout the storage period. The pH was measured using a calibrated SevenMulti™ pH meter (Mettler Toledo, Cornellà de Llobregat, Spain). A pH value between 7.3 and 8.3 was considered compliant with the specification [38]. Osmolality was measured with a calibrated Vapro^TM^ osmometer (Wescor, Inc., Logan, UT, USA). Particle and crystal detection was facilitated by a visual inspection station featuring black/white backgrounds under bright-field illumination, complemented by a SediMAX2™ phase-contrast microscope (77 Elektronika, Budapest, Hungary).

### 2.5. Microbiological Stability

Microbiological stability was assessed on the evaluation day (day 7 post-refrigerated storage following thawing), after simulating the use conditions of an opened eye drop in the RSG from day 1, 2, 3 and 7. On each day of analysis, between 08:00 h and 15:00 h (08:00 h, 10:00 h, 12:00 h, and 14:00 h), the eye drops were removed from the refrigerator and allowed to reach room temperature (approximately 15–20 min). The operator then washed and dried their hands, opened the container, and dispensed two drops. The container was subsequently closed and returned to the refrigerator.

The sample was inoculated into a culture medium for aerobic bacteria and fungi (BD BACTEC™ Peds Plus™/F, Becton Dickinson and Company, San Agustín de Guadalix, Spain), and a dedicated medium for anaerobic bacteria (BD BACTEC™ Lytic/10 Anaerobic/F, Becton Dickinson and Company, San Agustín de Guadalix, Spain). The culture media were incubated using the BD BACTEC™ fluorescent series system, which maintains a standardized incubation temperature of 35 °C ± 1.5 °C for 5 days in a CO_2_-containing atmosphere. The system detects microbial metabolism through CO_2_ production by the microorganisms present. Each vial contains a chemical sensor that detects increases in CO_2_ resulting from microbial growth. The sensor is monitored by the instrument every ten minutes for an increase in fluorescence, which is proportional to the amount of CO_2_ present. A positive result indicates the presumptive presence of viable microorganisms in the vial. The sensor does not appear visibly different between positive and negative vials; rather, it is the instrument that determines the difference through fluorescence. A vial is considered negative if, upon completion of the full 5-day protocol, the instrument has not detected a significant increase in fluorescence (CO_2_) and the vial shows no visual signs of positivity (turbidity, hemolysis, or septum bulging). Each box of these culture media is supplied with its own quality control certificates listing the test microorganisms, including ATCC^®^ strains specified in the CLSI M22 standard (Quality Control for Commercially Prepared Microbiological Culture Media), among them, *Streptococcus pyogenes* ATCC^®^ 19615, *Escherichia coli* ATCC^®^ 25922, *Streptococcus pneumoniae* ATCC^®^ 6305, *Pseudomonas aeruginosa* ATCC^®^ 27853, *Candida albicans* ATCC^®^ 18804, *Neisseria meningitidis* ATCC^®^ 13090, *Alcaligenes faecalis* ATCC^®^ 8750, *Haemophilus influenzae* ATCC^®^ 19418, and *Staphylococcus aureus* ATCC^®^ 25923.

## 3. Results

### 3.1. Validation of the Analytical Method

The HPLC method exhibited excellent linearity across the tested range, with a coefficient of determination (R2) = 0.9997 and a regression equation of y = 29,660,826x + 8,019.919 (Supplementary Materials Table S1 and Figure S1). Intra-day and inter-day precision for three meropenem quality control levels were highly satisfactory: intra-day relative standard deviation (RSD%) ranged from 0.114 to 0.150 (Table 1) and inter-day RSD% from 0.649 to 0.888 (Table 2), meeting the ICH criteria of ≤1% and ≤2% for repeatability and intermediate precision, respectively (Supplementary Materials Tables S2 and S3). Accuracy, determined by recovery rates at three concentration levels, ranged from 99.89% to 100.09%, falling within the accepted 98–102% interval (Supplementary Materials Table S4). The LOD and LOQ were calculated as 0.81 mg·mL^−1^ and 2.70 mg·mL^−1^, respectively.

### 3.2. Stability Study

#### 3.2.1. Freezing Conditions

**Figure 4:**
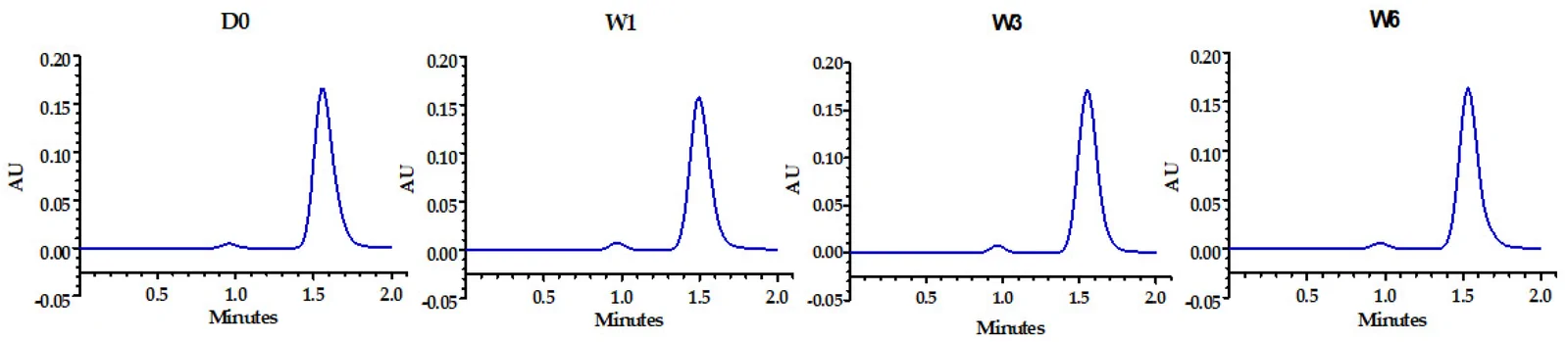
Chromatograms of meropenem 50 mg/mL eye drops in polypropylene dropper bottles under freezing conditions.

**Figure 5:**
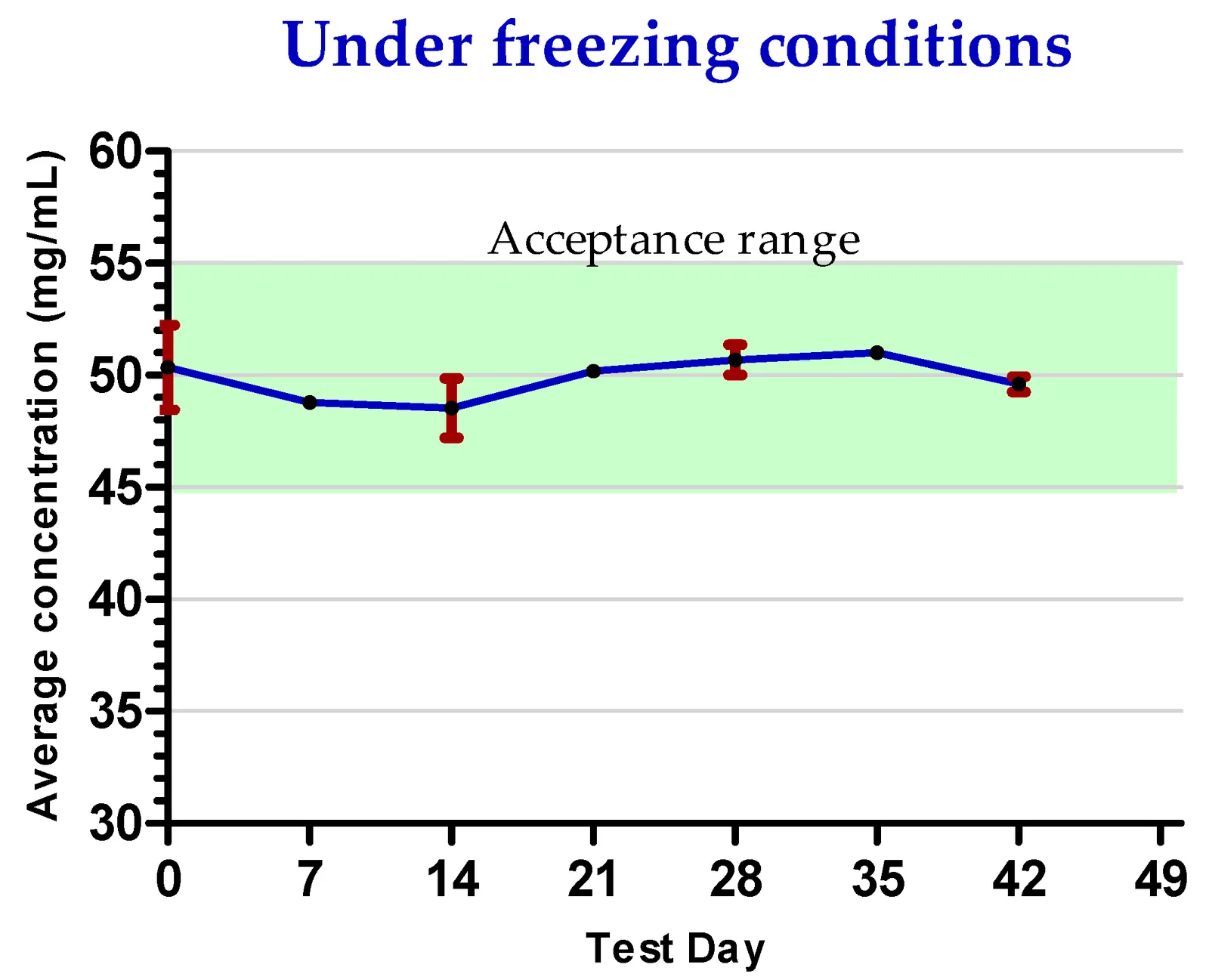
Evolution of the meropenem eye drop concentration in polypropylene dropper bottles under freezing conditions (nominal concentration 50 mg/mL).

The chemical stability assessment was performed by determining meropenem concentrations in the PP dropper bottle, on each scheduled sampling day, following the previously described procedure. Mean concentrations were calculated and reported as percentage recovery relative to the initial measurement (D0 = 100%), as shown in Table 3. Representative chromatograms from day 0 (D0) and the first day of the start of weeks 1, 3 and 6 (W1, W3 and W6) for the formulation under freezing conditions are shown in Figure 4. Results show that the concentration remained within the predefined acceptance limits throughout the six-week study period under freezing conditions (Table 3 and Figure 5). Detailed week-by-week concentration data are provided in the Supplementary Materials (Table S5).

Throughout the study, visual appearance (color and turbidity), visible particulates, crystallization and osmolality remained unchanged and the pH was within the acceptable range (Table 3).

Therefore, the 50 mg/mL meropenem ophthalmic solution in PP bottles, under freezing conditions, was considered physically and chemically stable for up to 42 days.

#### 3.2.2. Refrigerated Conditions

**Figure 6:**
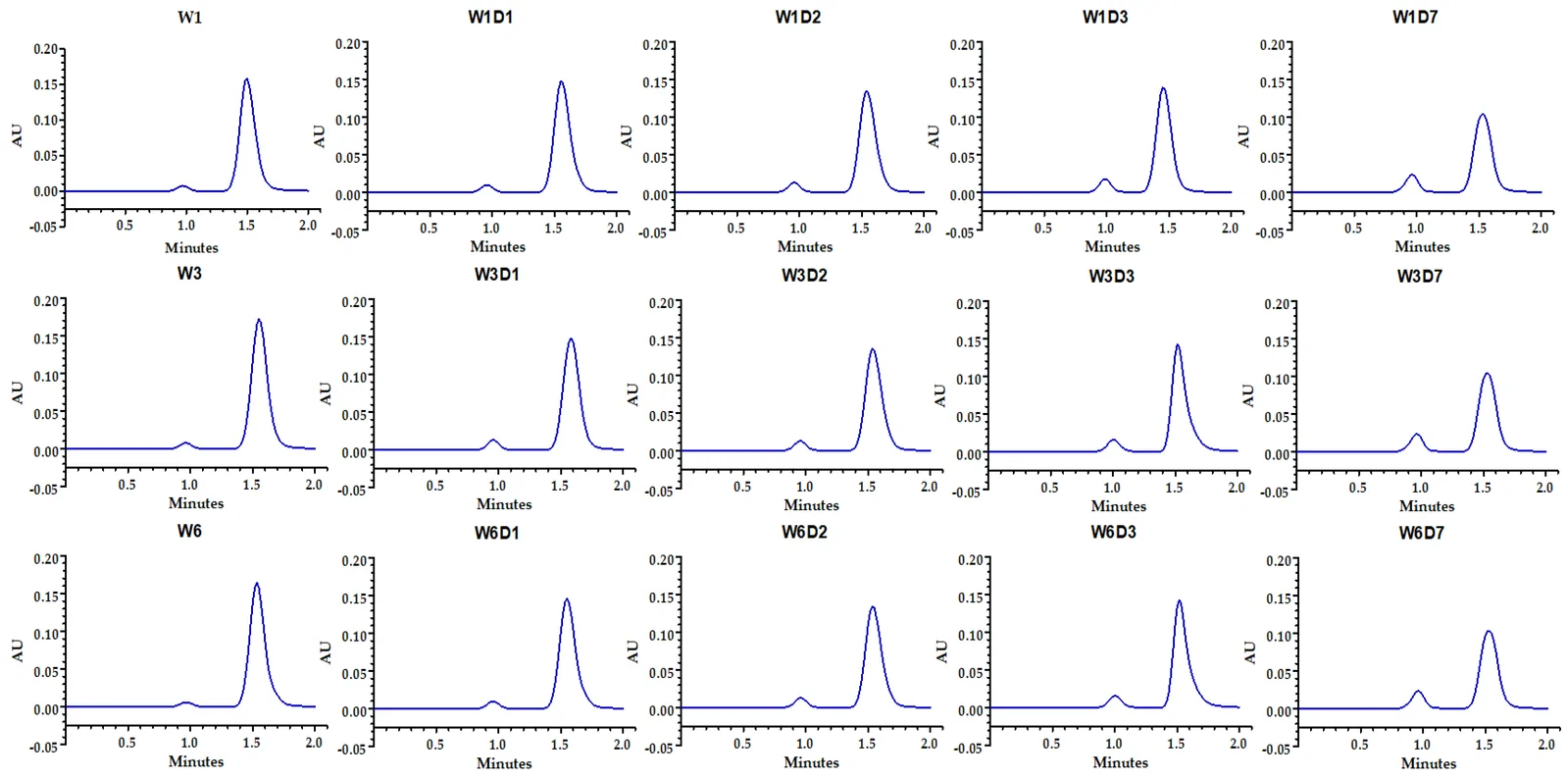
Chromatograms of meropenem 50 mg/mL eye drops in polypropylene dropper bottles under refrigerated conditions (W = week; D = day).

**Figure 7:**
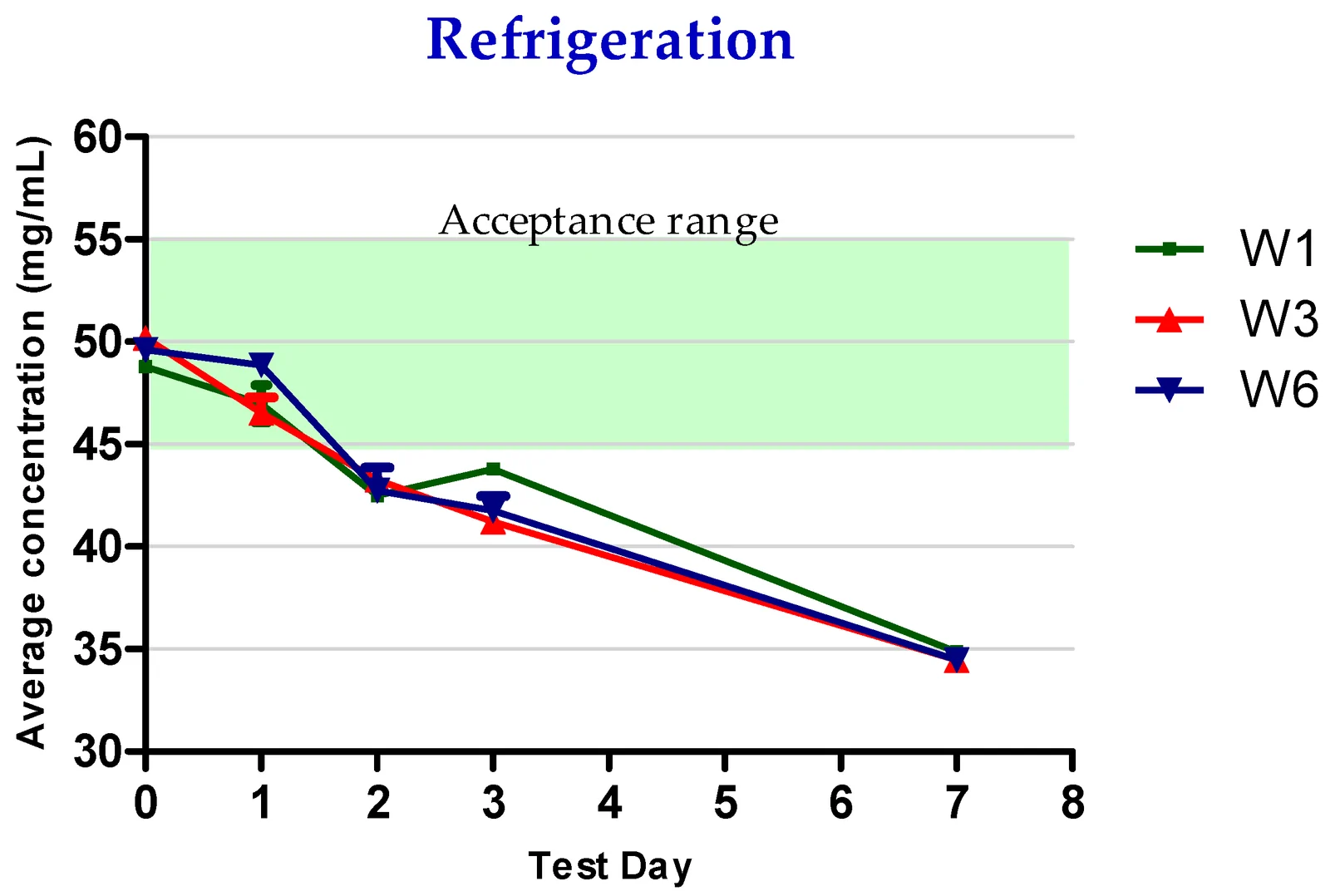
Evolution of the meropenem eye drop concentration in polypropylene dropper bottles under refrigeration conditions (nominal concentration 50 mg/mL).

The stability data in Table 4 and the chromatograms in Figure 6 correspond with the results obtained from the eye drops that were stored under freezing conditions for up to 1, 2, 3, 4, 5 and 6 weeks and after thawing, kept under refrigeration for 7 days, simulating real-life use conditions. For illustrative purposes, only W1, W3, and W6 of freezing are presented, followed by the chromatograms corresponding to days 1 (D1), 2 (D2), 3 (D3), and 7 (D7), during which the eye drops were stored under refrigerated conditions. Detailed concentration data (week-by-week and day-by-day of each week) are provided in the Supplementary Materials (Table S6). During this phase of the study, the concentration observed decreased progressively from the first day of refrigeration (Table 4 and Figure 7). Between days 1 and 2 of refrigeration, meropenem concentration reached the acceptance limit (45 mg/mL) relative to the nominal concentration of 50 mg/mL, i.e., it fell below 90% of the nominal value.

**Figure 8:**
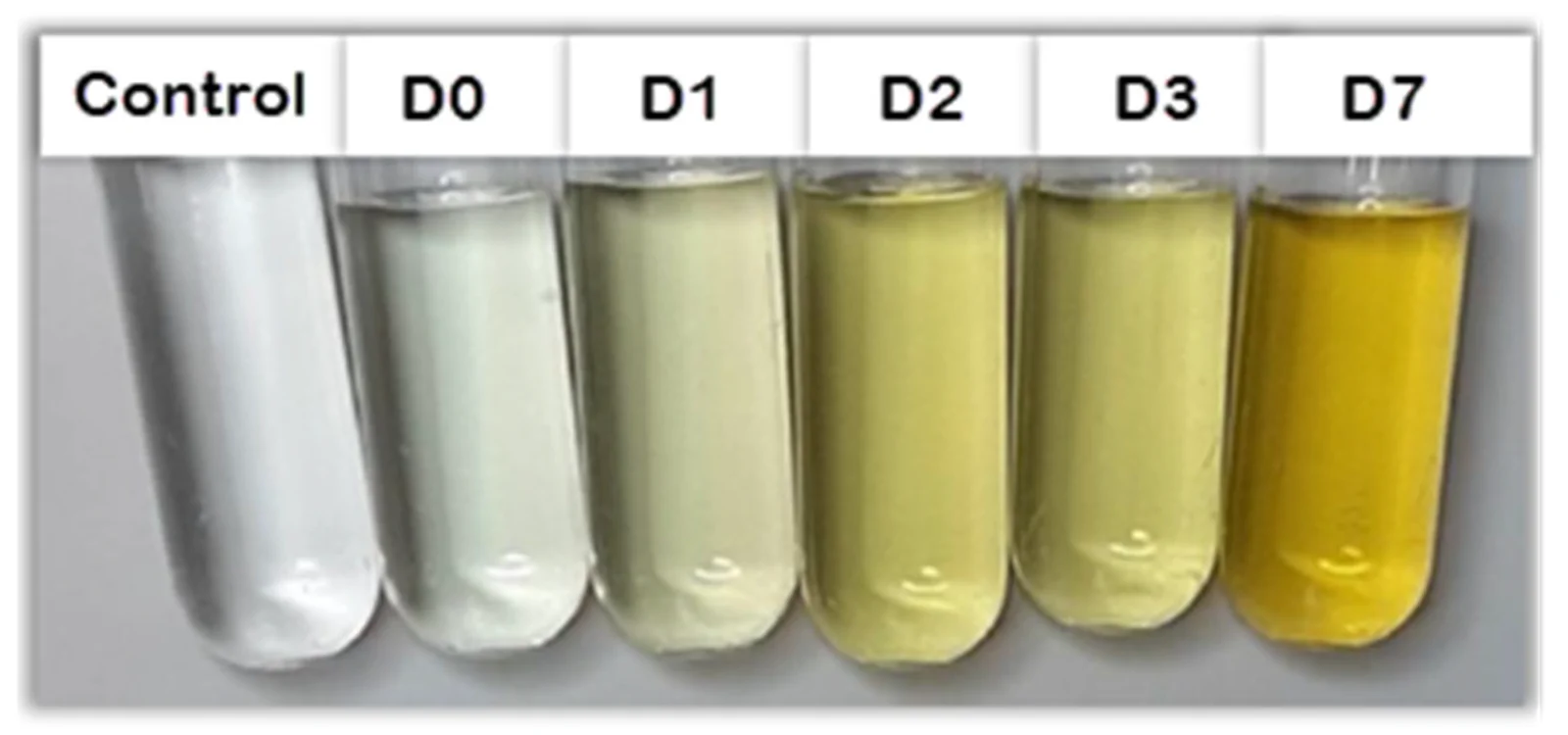
Color evolution of meropenem 50 mg/mL eye drops in a polypropylene dropper bottles during storage.

No significant changes were monitored in pH, osmolality, visible particulates, turbidity and crystallization throughout the study, but a progressive change in solution color was observed, evolving from colorless in the freshly prepared solution to a very pale yellow in the thawed samples subsequently stored under refrigeration, with the yellow color intensifying to a deeper shade by the last day (D7) of the refrigeration phase (Figure 8). This color change was reflected by a progressive increase in the chromatographic peak observed at approximately 1 min.

On day 7, aerobic, anaerobic, and fungal cultures of the 50 mg/mL meropenem eye drops stored in refrigerated PP bottles yielded negative results for all analyzed samples.

Thus, the meropenem 50 mg/mL eye drops in PP dropper bottles were regarded as physically and chemically stable for up to 24 h and microbiologically stable for up to 7 days under refrigeration conditions.

## 4. Discussion

The HPLC method for meropenem determination was linear, precise, accurate, repeatable, and reproducible, and was validated in accordance with ICH guidelines. The method was demonstrated to be robust and to reliably determine the kinetics run of meropenem in the presence of its degradation products, being consistent with previously published studies [34,39,40,41].

Regarding chemical stability, there are currently no stability studies of a meropenem eye drops at a concentration of 50 mg/mL frozen in a PP dropper bottle for the treatment of keratitis and endophthalmitis caused by multidrug-resistant G− bacteria [42]. Only one published study has evaluated a 50 mg/mL meropenem solution prepared from an injectable formulation in WFI, NS and D5W, stored in glass vials under two conditions: room temperature (21–26 °C and 23–27 °C) and refrigerated storage (4 °C) protected from the light. The study concluded that meropenem 50 mg/mL remained stable in WFI and NS for up to 8 h at 21–26 °C and up to 7 h at 23–27 °C in WFI; in D5W the stability was 3 h at 21–26 °C. Under refrigerated conditions, the stability was 48 h in WFI and NS, and 24 h in D5W [43]. This study was classified as level D evidence [44], with the following comments: “Stability indicating capability inadequately assessed, repeatability/reproducibility/standard range: results not provided or results outside specified values, no visual inspection, and no comments on degradation products” [42].

A total of five studies evaluating the stability of meropenem in PP containers have been published; specifically, in PP syringes. Of these, three assessed the stability at a concentration near to that of the present study. Two of them evaluated the stability of a 40 mg/mL meropenem solution in NS at room temperature (25 °C); one was not protected from the light and the other did not specify this condition, concluding that the stability was 4 and 8 h, respectively. The first study, in the order mentioned, was assigned a level of evidence of D, with the comment: “Repeatability/reproducibility/standard range: results not provided or results outside specified values” [45]. The second study was classified as level B evidence [46]. The third study evaluated the stability of a 41.7 mg/mL meropenem solution in NS and D5W at room temperature (20–25 °C) without light protection, with a level of evidence of C+, concluding that, at the aforementioned concentration, the meropenem solution is stable for up to 8 h in NS and 4 h in D5W [47].

It is not possible to compare the results of the first phase of this study under freezing conditions to those of other studies, as to date there are no published studies evaluating the stability of a 50 mg/mL meropenem ophthalmic solution under those conditions. With regard to refrigerated storage, although the studies performed were carried out in glass vials, the results of the present study indicate that the stability of the eye drops is lower than that reported in the published studies to date. This may perhaps be influenced by the fact that the refrigerated eye drops were subjected to temperature changes in order to simulate real-life administration conditions, which would involve removing the eye drops from the refrigerator, allowing them to equilibrate to room temperature, administering the prescribed drops, and then storing them again under refrigeration until the next administration. In any case, the result obtained would be aligned with the previously discussed studies, in which a 50 mg/mL meropenem solution in WFI in glass vials was stable for 7 h when stored at room temperature (23–27 °C) and for up to 48 h under refrigeration (4 °C). For practical purposes, the present study considered stability under these conditions to be 24 h, although, as shown in Figure 7, the acceptance limit (a decrease in concentration below 90% of the nominal value) would lie between 24 and 48 h.

With regard to the color change observed in the eye drops during refrigerated storage, this phenomenon is likely to be related to the hydrolytic cleavage of the beta-lactam ring. As previously mentioned in the introduction, this mechanism represents the primary degradation pathway of meropenem in aqueous solution. This ring-opening yields a more polar species with a shorter chromatographic retention time than intact meropenem. This finding may be associated with the peak observed at a retention time of 1 min, the area of which appears to increase in proportion to the intensification of the yellow coloration of the eye drops under refrigerated storage conditions. Further studies would be required to obtain conclusive results in this regard.

An important point to bear in mind is the osmolality of the eye drops evaluated in this study, which ranged from 325 to 326.750 mOsm/kg—slightly hyperosmolar relative to tear osmolality (290–310 mOsm/kg). The clinical significance of administering a hyperosmolar eye drop must be considered, both with regard to patient tolerability of the treatment and its potential toxicity to a compromised cornea.

Internationally, it is accepted that symptoms of acute irritation and epithelial cell damage (activation of inflammatory cascades) become consistent and significant once osmolality reaches or exceeds 340 mOsm/kg [48]. An osmolality of between 340 and 360 mOsm/kg is considered to induce osmotic stress, activating corneal polymodal nociceptors and cold thermoreceptors (TRPM8), which the patient perceives as pain and stinging [49]. Specific studies investigating how the eye responds to different osmotic concentrations have found that increasing osmolality lowered the pain threshold, suggesting the eye tolerates levels of up to 320–330 mOsm/kg well, whereas beyond 340 mOsm/kg the subjective discomfort response increases exponentially [50]. Likewise, Lemp et al. also regard 340 mOsm/kg as the “comfort tolerance” limit, concluding that between 340 and 350 mOsm/kg, nerve endings are affected [51]. In the case of an infected corneal ulcer with exposed nerve endings, this would translate into an immediate pain signal being transmitted to the brain.

It can therefore be concluded that international consensus establishes the 340 mOsm/kg threshold as the breaking point of ocular homeostasis and the onset of ocular damage [49]. The 50 mg/mL meropenem aqueous eye drop formulation does not reach the 340 mOsm/kg threshold, and good tolerability would therefore be expected; nevertheless, mild and transient adverse effects cannot be ruled out. The most frequent of these are a stinging or burning sensation, blurred vision, tearing, and foreign body sensation, comparable to those reported for other fortified antibiotic eye drops such as vancomycin 50 mg/mL or ceftazidime 50 mg/mL [52,53,54,55].

In terms of the main limitations of this study, it would have been very interesting and advisable to perform in vitro microbiological tests on the meropenem eye drops at D0 and D43, calculating the minimum inhibitory concentration (MIC) against the most frequent antibiotic-resistant bacteria involved in keratitis and endophthalmitis. Likewise, it would have been useful to verify whether meropenem adsorption to the polypropylene container wall occurs and whether it may contribute to the chemical instability of the refrigerated eye drops between 24 and 48 h.

Another point to consider is that formal forced-degradation studies were not performed in the present work; the method was adapted from a previously reported HPLC assay for meropenem and reoptimized under modified chromatographic conditions, including a different instrument, column brand, and column length, while keeping the mobile-phase composition unchanged. Under these conditions, the method proved adequate for the quantification of intact meropenem in the studied samples. Nevertheless, a dedicated selectivity evaluation against degradation products should be considered in future work to formally confirm its stability-indicating capability.

Although performing X-ray diffraction analysis or differential scanning calorimetry to evaluate the crystalline state of the drug during storage would have strengthened the consistency of our results, these techniques were unavailable at the time of the study.

In the present study, we used commercially available 1 g meropenem vials, which contain anhydrous sodium carbonate as an excipient. This point should be taken into account, as the results of this study would not be directly extrapolable if other commercial brands containing different excipients for the preparation of the eye drops were used.

## 5. Conclusions

The findings of this study demonstrate that 50 mg/mL meropenem eye drops in PP dropper bottles remain physicochemically stable for up to 42 days under freezing conditions (before opening), and for an additional 24 h after thawing when stored under refrigeration (after opening), for a total combined stability period of 43 days, protected from the light throughout. Furthermore, the ophthalmic solution maintained microbiological stability throughout this entire period.

## Figures and Tables

**Table 1:** Intra-day repeatability of meropenem eye drop samples at 80, 100 and 120% of the target concentration of 50 mg/mL.

Theoretical Concentration (mg/mL)	40 (80%)	50 (100%)	60 (120%)
Mean (mg/mL)	39.903	49.892	59.940
SD	0.06	0.06	0.068
RSD%	0.150	0.120	0.114
Accuracy%	99.758	99.783	99.899

RSD%: Relative standard deviation; SD = Standard deviation.

**Table 2:** Inter-day repeatability of meropenem eye drop samples at 80, 100 and 120% of the target concentration of 50 mg/mL.

Theoretical Concentration (mg/mL)	40 (80%)	50 (100%)	60 (120%)
Mean (mg/mL)	40.073	50.143	60.017
SD	0.356	0.337	0.389
RSD%	0.888	0.671	0.649
Accuracy%	100.182	100.285	100.029

RSD%: Relative standard deviation; SD = Standard deviation.

**Table 3:** Physicochemical results of meropenem 50 mg/mL eye drops in polypropylene dropper bottles under freezing conditions.

Parameter	D0	W1	W3	W6
AVR%	100	97.554 ± 0.068	100.367 ± 0.219	99.211 ± 0.266
pH	7.987 ± 0.078	7.957 ± 0.110	8.045 ± 0.030	8.202 ± 0.062
Osmolality (mOsm/kg)	326.750 ± 2.754	NM	NM	326.250 ± 1.708
Color	Very pale yellow	Very pale yellow	Very pale yellow	Very pale yellow
Visible Particulates	Absence	Absence	Absence	Absence
Crystals ≥ 10 µm/mL	0	0	0	0

Results are expressed as mean ± SD (standard deviation) of triplicate determinations; AVR% = Average recovery percentage; D0 = Day 0 of the test; NM = Not measured; W1 = First week of the test; W3 = Third week of the test; W6 = Sixth week of the test.

**Table 4:** Physicochemical results of meropenem 50 mg/mL eye drops in polypropylene drop bottles under refrigeration conditions, after thawing.

Code	Week	Test Day	SC	AVR%	Osm	pH	Color	Visible Particulates	Crystals ≥ 10 µm/mL
		0		100	326.750 ± 2754	7.987 ± 0.078	Very pale yellow	Absence	0
W1	1	7	Frozen	97.554 ± 0.068	NM	7.957 ± 0.110	Very pale yellow	Absence	0
W1D1	1	8	Cooled	93.945 ± 0.001	NM	8.092 ± 0.021	Light pale yellow	Absence	0
W1D2	1	9	Cooled	84.959 ± 0.001	NM	8.085 ± 0.017	Pale yellow	Absence	0
W1D3	1	10	Cooled	87.597 ± 0.001	NM	7.942 ± 0.013	Moderate yellow	Absence	0
W1D7	1	13	Cooled	69.723 ± 0.001	NM	7.903 ± 0.042	Intense yellow	Absence	0
W3	3	21	Frozen	100.367 ± 0.219	NM	8.045 ± 0.030	Very pale yellow	Absence	0
W3D1	3	22	Cooled	93.076 ± 0.006	NM	8.129 ± 0.022	Light pale yellow	Absence	0
W3D2	3	23	Cooled	86.533 ± 0.001	NM	8.097 ± 0.026	Pale yellow	Absence	0
W3D3	3	24	Cooled	82.426 ± 0.002	NM	7.931 ± 0.033	Moderate yellow	Absence	0
W3D7	3	27	Cooled	68.928 ± 0.004	NM	7.861 ± 0.020	Intense yellow	Absence	0
W6	6	42	Frozen	99.211 ± 0.266	326.250 ± 1708	8.202 ± 0.062	Very pale yellow	Absence	0
W6D1	6	43	Cooled	97.744 ± 0.001	325.000 ± 1414	8.100 ± 0.012	Light pale yellow	Absence	0
W6D2	6	44	Cooled	85.494 ± 0.009	NM	8.078 ± 0.017	Pale yellow	Absence	0
W6D3	6	45	Cooled	83.539 ± 0.006	NM	7.971 ± 0.024	Moderate yellow	Absence	0
W6D7	6	48	Cooled	68.900 ± 0.004	NM	7.845 ± 0.044	Intense yellow	Absence	0

Results are expressed as mean ± SD (standard deviation) of triplicate determinations; AVR% = Average recovery percentage; D0 = Day 0 of the test; D1, D2, D3 and D7 = Day 1, 2, 3 and 7 of the test under refrigeration; NM = Not measured; Osm = Osmolality (mOsm/kg); SC = Storage condition; W1 = First week of the test; W3 = Third week of the test; W6 = Sixth week of the test.

## Data Availability

The data presented in this study are available on request from the corresponding author.

## Supplementary Materials

The following supporting information can be downloaded at https://www.mdpi.com/article/10.3390/pharmaceutics18080971/s1, Table S1: Peak area and the meropenem concentration for making the calibration curve; Table S2: Data from intra-day repeatability of meropenem samples at 80, 100 and 120% of the target concentration of 50 mg/mL; Table S3: Data from inter-day repeatability of meropenem samples at 80, 100 and 120% of the target concentration of 50 mg/mL; Table S4: Data from accuracy of meropenem samples at 80, 100 and 120% of the target concentration of 50 mg/mL; Table S5: Average Recovery% of three determinations on the day of the test and pH of meropenem 50 mg/mL eye drops in PP dropper bottles under freezing conditions; Table S6: Average Recovery% of three determinations on the day of the test and pH of meropenem 50 mg/mL eye drops in PP dropper bottles under refrigerated conditions; Figure S1: Linearity between the peak area and the meropenem concentration for making the calibration curve.

## Abbreviations

The following abbreviations are used in this manuscript:

ASC	Anhydrous sodium carbonate
D5W	5% aqueous dextrose solution
ESBLs	Extended spectrum β-lactamases
FSG	Frozen stability group
G−	Gram-negative bacteria
G+	Gram-positive bacteria
GPPMHPS	Guide for good preparation practices of medicines in hospital pharmacy services
ICH	International Council for Harmonisation of Technical Requirements for Pharmaceuticals for Human Use
LOD	Limit of detection
LOQ	Limit of quantification
MIC	Minimum inhibitory concentration
NM	Not measured
NS	0.9% aqueous sodium chloride solution
PP	Polypropylene
RSD%	Relative standard deviation
RSG	Refrigerated stability group
SC	Storage conditions
WFI	Water for Injection
