# Supplementary material for: Stability Study of Meropenem 50 mg/mL Eye Drops in Polypropylene Dropper Bottles
Source: Pharmaceutics. 2026 Aug 7;18(8):971. doi: 10.3390/pharmaceutics18080971 (PMC13516646; doi:10.3390/pharmaceutics18080971)
Supplement: Supplementary file 1 [file pharmaceutics-18-00971-s001.zip › pharmaceutics-4450403-supplementary.pdf]

Supplementary Material

Supplementary Table S1: Peak area and the meropenem points concentrations for making the calibration curve.

Point	Concentration (mg/mL)	Peak area (µV*sec)
1	0	2.480E+03
1	0	3,004E+02
1	0	7,005E+01
1	0	6,342E+00
1	0	9,295E+00
1	0	4,579E+02
2	15	4,525E+05
2	15	4,580E+05
2	15	4,597E+05
2	15	4,572E+05
2	15	4,578E+05
2	15	4,580E+05
3	30	8,126E+05
3	30	9,169E+05
3	30	8,547E+05
3	30	9,090E+05
3	30	9,136E+05
3	30	8,993E+05
4	60	1.786E+06
4	60	1,786E+06
4	60	1,810E+06
4	60	1,821E+06
4	60	1,818E+06
4	60	1,791E+06
5	90	2,716E+06
5	90	2,692E+06
5	90	2,679E+06
5	90	2,695E+06
5	90	2,684E+06
5	90	2,713E+06
6	120	3,583E+06
6	120	3,610E+06
6	120	3,516E+06
6	120	3,503E+06
6	120	3,547E+06
6	120	3,597E+06
7	150	4.405E+06
7	150	4,468E+06
7	150	4,457E+06
7	150	4,469E+06
7	150	4,447E+06
7	150	4,447E+06

Supplementary Figure S1: Linearity between the peak area and the Meropenem 50 mg/mL eye drops concentration for making the calibration curve.

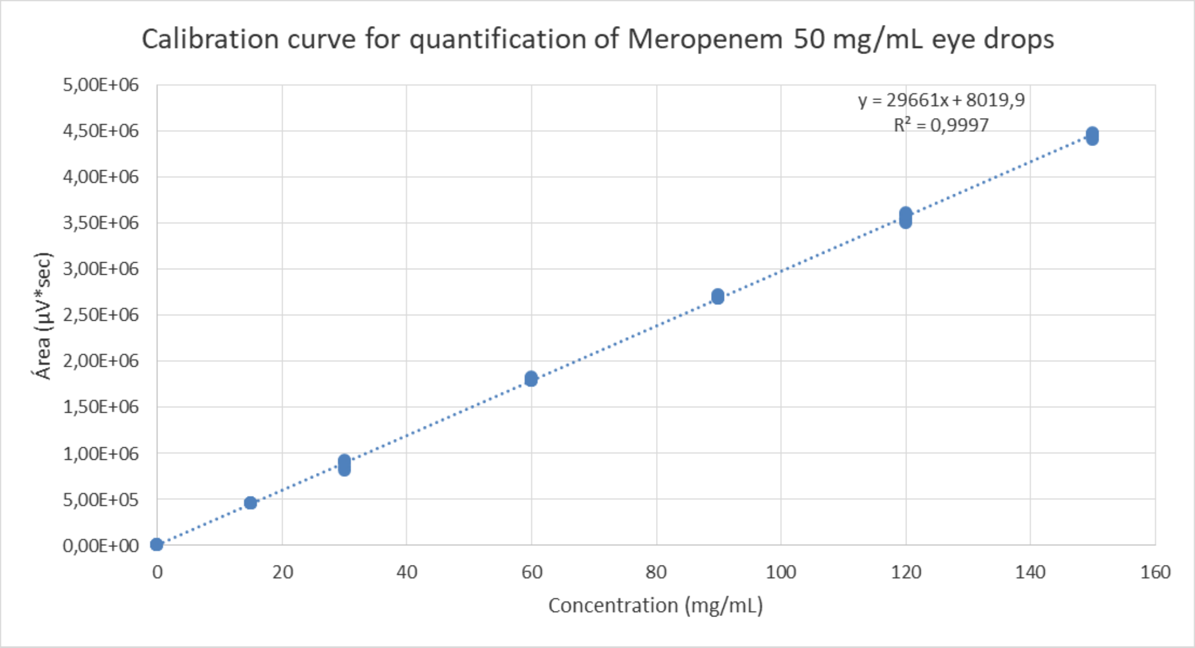

Supplementary Table S2: Data from intra-day repeatability of meropenem 50 mg/mL eye drop samples at 80, 100 and 120% of the target concentration (50 mg/mL)

Target concentration (mg/mL)	Estimated concentration (mg/mL)	Mean	SD	RSD%	RSD% mean	Accuracy%	Accuracy% mean
	39.851					99.628	
	39.909					99.773	
40 (80%)	39.992	39.903	0.060	0.150		99.980	99.758
	39.831					99.578	
	39.947					99.867	
	39.888					99.720	
	49.806					99.612	
	49.886					99.771	
50 (100%)	49.973	49.892	0.060	0.120	0.128	99.945	99.783
	49.846					99.692	
	49.931					99.862	
	49.908					99.817	
	59.843					99.739	
	59.943					99.906	
60 (120%)	60.038	59.940	0.068	0.114		100.063	99.899
	59.897					99.828	
	59.989					99.982	
	59.927					99.878	

RSD%: Relative standard deviation ; SD= Standard deviation

Supplementary Table S3: Data from inter-day repeatability of Meropenem 50 mg/mL eye drops samples at 80, 100 and 120% of the target concentration (50 mg//mL

Target concentration (mg/mL)	Estimated concentration (mg/mL)	Day	Mean	SD	RSD (%)	RSD% mean	Accuracy %	Accuracy% mean
40 (80%)	40.391	1	40.073	0.356	0.888	0.736	100.978	100.182
	40.596	1					101.490	
	40.619	1					101.548	
	39.978	2					99.945	
	40.025	2					100.063	
	39.842	2					99.605	
	39.847	3					99.618	
	40.054	3					100.135	
	39.86	3					99.650	
	39.925	4					99.813	
	39.576	4					98.940	
	39.443	4					98.608	
	40.319	5					100.798	
	40.534	5					101.335	
	40.081	5					100.203	
50 (100%)	50.488	1	50.143	0.337	0.671		100.976	100.285
	50.745	1					101.490	
	50.773	1					101.546	
	50.006	2					100.012	
	50.319	2					100.638	
	50.054	2					100.108	
	49.978	3					99.956	
	50.006	3					100.012	
	49.923	3					99.846	
	49.841	4					99.682	
	49.628	4					99.256	
	49.738	4					99.476	
	50.113	5					100.226	
	50.319	5					100.638	
	50.208	5					100.416	
60 (120%)	59.32	1	60.017	0.389	0.649		98.867	100.029
	59.488	1					99.147	
	59.621	1					99.368	
	59.744	2					99.573	
	59.831	2					99.718	
	59.905	2					99.842	
	59.962	3					99.937	
	60.004	3					100.007	
	60.053	3					100.088	
	60.118	4					100.197	
	60.208	4					100.347	
	60.311	4					100.518	
	60.428	5					100.713	
	60.566	5					100.943	
	60.703	5					101.172	

RSD%: Relative standard deviation; SD= Standard deviation

Supplementary Table S4: Data from accuracy of Meropenem 50 mg/mL eye drop samples at 80, 100 and 120% of the target concentration of 50 mg/mL (recovery expressed as a percentage of recovery of the theoretical value -100%-)

Target concentration (mg/mL)	Estimated concentration (mg/mL)	Recovery%	Mean	SD	RSD%
40(80%)	39.974	99.936	99.982	0.163	0.163
	39.956	99.890			
	40.060	100.150			
	39.984	99.960			
	40.112	100.280			
	40.128	100.320			
50 (100%)	49.960	99.919			
	49.833	99.666			
	49.948	99.896			
	49.871	99.742			
	49.998	99.997			
	50.058	100.117			
60 (120%)	60.008	100.014			
	59.951	99.918			
	60.022	100.037			
	59.997	99.995			
	59.993	99.988			
	59.913	99.856			

Recovery%= Percentage of recovery of the theoretical value (100%); RSD%; Relative standard deviation; SD = Standard deviation;

Supplementary Table S5: Average Recovery% of three determinations on the day of the test and pH of frozen Meropenem 50 mg/mL eye drops

Test Day	Week	C1 (mg/mL)	R% C1	C2 (mg/mL)	R% C2	C3 (mg/mL)	R% C3	AVR%	pH
0		49,78	99,555%	50,07	100,137%	51,22	102,437%	100,71%	7,987
8	W1	48,80	97,603%	48,74	97,480%	48,79	97,580%	97,55%	7,957
15	W2	48,73	97,468%	48,95	97,909%	47,93	95,864%	97,08%	7,965
22	W3	50,06	100,115%	50,25	100,507%	50,24	100,479%	100,37%	8,045
29	W4	50,84	101,681%	50,38	100,753%	50,85	101,703%	101,38%	8,083
36	W5	51,02	102,046%	50,98	101,953%	51,02	102,037%	102,01%	8,169
43	W6	49,76	99,514%	49,55	99,104%	49,51	99,016%	99,21%	8,202

R% = Recovery percentage; AVR% = Average recovery percentage of C1, C2 and C3; W = Weeks of freezing

Supplementary Table S6: Average Recovery% of three determinations on the day of the test and pH of ~~r~~ Meropenem 50 mg/mL eye drops under refrigerated conditions

Weak	Test Day	C1 (mg/mL)	Re% C1	C2 (mg/mL)	R% C2	C3 (mg/mL)	R% C3	AVR%	pH
W1	0	48,80	97,603%	48,74	97,480%	48,79	97,580%	97,554%	7.957
W1	1	47,26	94,518%	46,56	93,116%	47,10	94,201%	93,945%	8.092
W1	2	42,57	85,142%	42,45	84,907%	42,41	84,829%	84,959%	8.085
W1	3	43,80	87,604%	43,82	87,631%	43,78	87,556%	87,597%	7.942
W1	7	34,86	69,723%	34,86	69,723%	34,86	69,723%	69,723%	7.903
W2	0	48,73	97,468%	48,95	97,909%	47,93	95,864%	97,080%	7.965
W2	1	46,34	92,687%	46,98	93,955%	47,03	94,063%	93,568%	8.094
W2	2	43,06	86,118%	42,83	85,663%	42,70	85,391%	85,724%	8.074
W2	3	42,00	84,008%	42,08	84,150%	42,11	84,221%	84,126%	8.012
W2	7	34,25	68,499%	34,32	68,630%	34,33	68,660%	68,596%	7.845
W3	0	50,06	100,115%	50,25	100,507%	50,24	100,479%	100,367%	8.045
W3	1	46,29	92,581%	46,87	93,742%	46,45	92,904%	93,076%	8.129
W3	2	43,27	86,533%	43,27	86,533%	43,27	86,533%	86,533%	8.097
W3	3	41,29	82,588%	41,09	82,182%	41,25	82,508%	82,426%	7.931
W3	7	34,24	68,476%	34,69	69,381%	34,46	68,927%	68,928%	7.861
W4	0	50,84	101,681%	50,38	100,753%	50,85	101,703%	101,379%	8.083
W4	1	46,31	92,612%	46,31	92,612%	46,34	92,686%	92,637%	8.101
W4	2	42,47	84,948%	42,51	85,025%	42,44	84,874%	84,949%	8.073
W4	3	41,46	82,913%	41,74	83,476%	42,05	84,108%	83,499%	8.004
W4	7	34,34	68,672%	34,74	69,489%	34,48	68,955%	69,039%	7.889
W5	0	51,02	102,046%	50,98	101,953%	51,02	102,037%	102,012%	8.169
W5	1	47,43	94,862%	47,44	94,872%	47,48	94,966%	94,900%	8.070
W5	2	43,14	86,274%	42,51	85,018%	42,97	85,947%	85,746%	8.081
W5	3	41,52	83,041%	41,33	82,667%	41,95	83,892%	83,200%	7.989
W5	7	34,56	69,118%	34,38	68,764%	34,80	69,602%	69,161%	7.902
W6	0	49,76	99,514%	49,55	99,104%	49,51	99,016%	99,211%	8.202
W6	1	48,94	97,886%	48,83	97,669%	48,84	97,676%	97,744%	8.100
W6	2	42,38	84,756%	43,25	86,501%	42,61	85,225%	85,494%	8.078
W6	3	41,81	83,614%	42,03	84,057%	41,47	82,945%	83,539%	7.971
W6	7	34,46	68,913%	34,62	69,247%	34,27	68,541%	68,900%	7.845
